# Current understanding of genetic polymorphisms as biomarkers for risk of biological complications in implantology

**DOI:** 10.4317/jced.55141

**Published:** 2018-10-01

**Authors:** Asier Eguia del Valle, José López-Vicente, Rafael Martínez-Conde, Luis-Antonio Aguirre-Zorzano

**Affiliations:** 1 Associate Professor. DDS, PhD.Departamento Estomatología II, Facultad de Medicina y Enfermería. Universidad del País Vasco (UPV/EHU). // Stomatology II Department, Faculty of Medicine and Nursering. University of the Basque country (UPV/EHU). Leioa (Vizcaya) Spain; 2 Associate Professor. MD, DDS, PhD, Departamento Estomatología II, Facultad de Medicina y Enfermería. Universidad del País Vasco (UPV/EHU). // Stomatology II Department, Faculty of Medicine and Nursering. University of the Basque country (UPV/EHU). Leioa (Vizcaya) Spain; 3 Professor. MD, DDS, PhD, Departamento Estomatología II, Facultad de Medicina y Enfermería. Universidad del País Vasco (UPV/EHU). // Stomatology II Department, Faculty of Medicine and Nursering. University of the Basque country (UPV/EHU). Leioa (Vizcaya) Spain; 4 Professor. MD,DDs, PhD. Departamento Estomatología II, Facultad de Medicina y Enfermería. Universidad del País Vasco (UPV/EHU). // Stomatology II Department, Faculty of Medicine and Nursering. University of the Basque country (UPV/EHU). Leioa (Vizcaya) Spain. Director of Master of Periodontology at the University of The Basque Country

## Abstract

**Background:**

In the last decade, multiple studies have been published that analyze the relationship between the risk of experiencing biological complications with implants and the presence of certain types of genetic polymorphisms. In the present report, we analyze the controversies that have arisen from this important area of investigation and synthesize the most prominent aspects of knowledge related to this possible etiopathogenic relationship.

**Material and Methods:**

For this review, the biomedical databases PubMed-Medline, SciELO, and DOAJ were used. Different search strategies were employed, from which 298 articles initially emerged. After refinement of the search, 55 articles published between 2002 and 2018 were finally selected based on relevance.

**Results:**

In certain population groups, there is evidence to support that about a dozen polymorphisms could in some way be related to biological complications in implantology. Indeed, the results may vary according to the ethnic origin of the population studied. Most of the published investigations are initial studies reporting small sample sizes and utilizing different study group homogenization methods. We are still at a preliminary stage of our understanding and development with regard to these types of biomarkers. The interesting results identified indicate that new investigations will be necessary to eliminate the biases observed in some studies and to homogenize the research groups. In order to clarify the controversies surrounding the current knowledge in this field, we believe that it will be necessary to employ larger study groups and search for possible synergistic effects between different polymorphisms.

** Key words:**Polymorphism, genetic markers, peri-implantitis, biological complication, dental implant.

## Introduction

Currently, implantology is a predictable and reproducible alternative for replacing missing teeth. Improvements in the design and physical-chemical properties of implants and related treatment protocols have led to a drastic reduction in problems with osseointegration, which were initially observed in a significant number of cases. Indeed, the most recent studies have shown that the rate of early failure does not exceed 1-2% of cases (1,2). However, modern dental implantology is currently facing a challenge to reduce the increasing rates of peri-implant disease (2,3). Despite the discrepancies in diagnostic criteria and inclusion in different studies, more than 60% of individuals and 30% of implants show evidence of the presence of peri-implant mucositis, while up to 18% of patients and over 9% of the implants demonstrate some degree of peri-implantitis (3,4).

Several factors are known to play a role in the complex etiology of peri-implant disease, such as tobacco consumption, oral hygiene, the presence of periodontal disease, oral biofilm composition, bone quality, certain systemic pathologies (e.g., diabetes), implant type, the techniques employed for implant placement, prosthesis design or transmission of occlusal loads (5-8).

In a straightforward manner, all of these factors act through three different pathogenic mechanisms, which are: disruption of the balance between the biofilm and the patient’s own immune system, overcoming the repair capacity of bone tissue and modifications in implant surfaces resulting from corrosion (5-8). Each of these mechanisms can stimulate hyperproduction of proinflammatory cytokines and increase osteoclastic activity in tissues surrounding the implant. These phenomena promote rupture of the physiological balance between bone apposition and resorption in favor of a greater resorptive activity in the peri-implant bone tissue (4-8).

Of course, these etiopathogenic factors could be directly and indirectly conditioned by genetic factors that determine their magnitude and relevance in each individual. They are part of what we call “individual susceptibility” and explain the different biological responses observed in individuals presenting the same risk factors (5-8).

Epidemiological studies have shown the existence of patients having a “certain predisposition” or augmented risk of suffering from peri-implant disease. In these patients, failures tend to cluster or “clump together” (9). This observation has made numerous authors ask themselves whether such “predisposition” or “greater risk” could be, at least in part, genetically conditioned (9,10). Another question, which is equally as important if not more than the previous one, is whether “predisposition” could be detected or quantified. If so, we could know beforehand the relative risk of a particular patient to suffer from peri-implant disease due to their genetic characteristics or “peculiarities” and proactively establish personalized diagnostic and therapeutic protocols to reduce its prevalence (9,10).

These genetic “peculiarities” are caused by DNA mutations. Some of these mutations are significantly prevalent in the population (above 1%), constituting what are called “genetic polymorphisms”. For this reason, in recent years, several studies have been conducted to try to understand the relationship between certain polymorphisms and peri-implant disease. The majority of the polymorphisms studied affect a single nucleotide and are called SNPs (Single Nucleotide Polymorphism). Among these, in relation to peri-implant disease, those that are present in the genes involved in inflammatory responses and regulation of bone metabolism have been specifically studied (10).

The role of genetic polymorphisms in different autoimmune, neurological and even oncological pathologies has been widely studied (11,12). On the contrary, in the case of peri-implant disease, there are still few studies and the results remain inconclusive. However, the available studies are promising and justify additional efforts to continue investigating in this field. In this narrative review, we try to synthesize and perform a critical analysis on the relationship between genetic polymorphisms, peri-implant disease and the risk of early implant failure.

## Material and Methods

For this literature review, the biomedical databases PubMed-Medline, SciELO and DOAJ were used. Different search strategies were employed, resulting from the combination of keywords from the MeSH terminology dictionary such as: “polymorphism”, “genetic markers”, “genetic polymorphism”, “peri-implantitis”, “implant failure” and “dental implant”.

Using this initial search strategy, 298 articles were found. After the elimination of duplicate articles, followed by evaluation and filtering of the remaining articles, 55 relevant publications were finally selected, published between 2002 and 2018 Figure 1.

**Figure 1:**
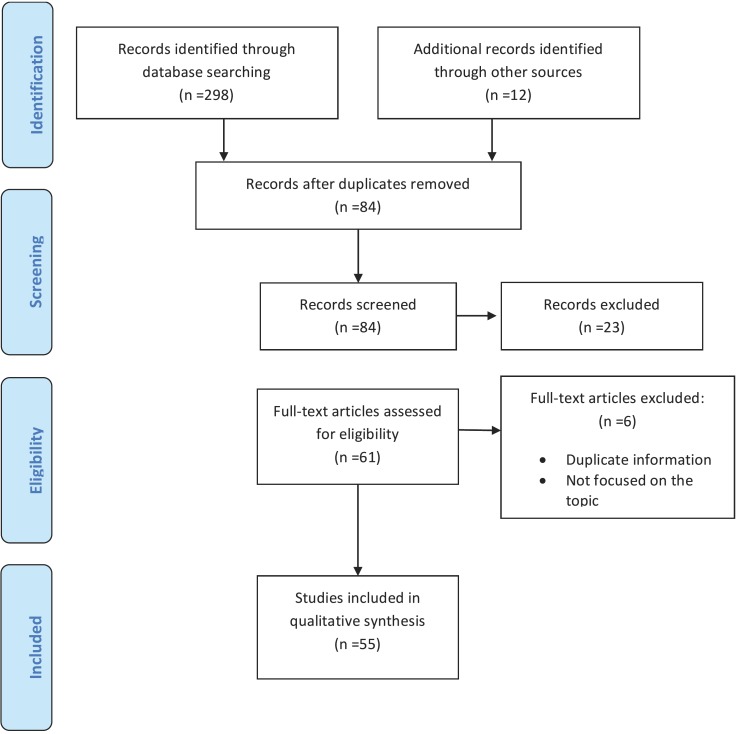
Article selection flow diagram. Adapted from: The PRISMA Group (2009). Preferred Reporting Items for Systematic Reviews and Meta-Analyses: The PRISMA Statement. PLoS Med 6(7): e1000097.

## Results

-Polymorphisms of IL-1 

Cytokines are signaling molecules responsible for intercellular communication that induces the activation of specific membrane receptors. Some of their functions contribute to control of the inflammatory response (13-15). For instance, interleukin-1 (IL-1), produced mainly by monocytes and activated macrophages, is a potent proinflammatory cytokine. This interleukin is not a single molecule, but a group that includes three different cytokines: IL-1α, IL-1β and the IL-1 receptor antagonist (IL-1Ra or IL-1RN). The latter actually functions as an anti-inflammatory cytokine because it binds non-productively to the IL-1 receptor, specifically acting as a natural inhibitor (13-16).

These proteins are encoded by three different genes: IL1A, IL1B, and IL1RN, which make up the IL-1 gene cluster, located on chromosome 2 (13,14,16). At the oral level, several studies (15-18) have demonstrated the presence of high levels of IL-1α and IL-1β in crevicular fluid and in saliva of patients with active peri-implantitis lesions. Moreover, it has been proven that the levels of these proinflammatory cytokines can even be reduced with treatment of the injuries. Also, it has been observed that with increased clinical severity or more advanced stage of peri-implant disease, IL-1 levels are higher and maintain a good correlation with parameters such as probing or plaque index (15-18). On the other hand, “de novo” plaque accumulation tests have revealed increased levels of IL-1 and Tumor Necrosis Factor (TNF), which is another proinflammatory cytokine, 30 days after brushing was stopped and subsequent decreased levels when hygiene was restored (16).

Some functionally relevant polymorphisms in the genes that encode IL-1, under certain circumstances, can condition overproduction and even a basal increase in production of the respective cytokines (13-16). For example, the presence of the nucleotide Thymine (T) in certain polymorphic regions of IL-1A (889-), IL-1B (+3954) or IL-1B (511-) can lead to an increased transcriptional activity of the IL1A and IL1B genes, constituting “positive” or at-risk alleles. This phenomenon has been linked to a wide range of disorders, in which there is an altered inflammatory response, such as rheumatoid arthritis, psoriasis or inflammatory bowel disease as well as other pathologies (e.g., cancer) (11,12). At the oral level, polymorphisms of IL-1 have been widely studied in relation to periodontal disease (19). More recently, an attempt has been made to identify whether these polymorphisms are related to an augmented risk for early dental implant failure or peri-implantitis (16-18).

Polymorphisms in the IL-1 cluster are the most researched in the scientific literature in relation to peri-implantitis and early implant failure. However, as we will analyze below, the results of these investigations have been somewhat contradictory. In this regard, the most studied polymorphic regions are IL-1A (889-), IL-1B (511-) and IL-1B (+3954) (20-25). Reports related to carriers of risk SNPs with early failure or peri-implantitis have shown different results. Indeed, some authors have demonstrated a greater relative risk in carrier patients (10,20-23). For instance, Cosyn *et al.* (20) analyzed a reduced sample size (14 patients with early failure and 14 controls) and observed an increased risk of early failure in carriers of the nucleotide Thymine (T) within regions of IL-1A (889-) (Odds Ratio OR = 3.9) and IL-1B (+3954) (OR = 15).

In an interesting study by Gruica *et al.* (25), analyzing tobacco consumption, the prevalence of biological complications and the IL-1 genotype of 180 patients with dental implants, a statistically significant synergistic effect was observed between the presence of a positive allele and being a severe smoker with an increased risk of suffering from peri-implant pathology.

Notably, when these SNPs are analyzed in combination instead of separately, the results are even more contradictory. In this regard, studies have examined the relationship between peri-implant pathology and carrying a combined genotype with SNPs IL-1A (889-) / IL-1B (+3954) or IL-1A (889-) / IL-1B (511-) / IL-1B (+3954). In this way, the meta-analysis conducted by Liao *et al.* (21) reported an increased risk of early failure (OR = 1.76) and peri-implantitis (OR = 2.34) in carriers with both IL-1A (889-) and IL-1B (+3953) positive alleles. These data undergo variations when adjusted for ethnic origin, decreasing for Caucasians and European descendants.

Given the great diversity of results in relation to IL-1 polymorphisms, an interesting review by Bormann *et al.* (15) divided the results obtained by different groups of researchers into four types: 1) those that do not find a statistically significant relationship between polymorphisms of IL-1 and peri-implantitis, 2) those concluding that there is an uncertain relationship, 3) those that show a significant relationship and 4) those that report a relationship only under the influence of certain added risk factors (e.g., tobacco use). Importantly, Bormann *et al.* conclude that there is a greater number of studies that describe a positive relationship, with or without the presence of other risk factors (15).

Recently, other authors (10,21,24) have published review and meta-analysis studies. For example, Huynh-Ba *et al.* (24) suggested that there is not sufficient evidence to contradict the existence of a relationship between IL-1 polymorphisms and the risk of peri-implantitis. However, Dereka *et al.* (10) concluded that there is only a certain tendency that can be appreciated that warrants further study for confirmation.

-Polymorphisms of TNFα

Tumor Necrosis Factor-α (TNFα) is another key proinflammatory cytokine in the early stages of the inflammatory response (26). Similar to IL-1, elevated TNFα levels have been observed in crevicular fluid and saliva of patients with active peri-implantitis lesions and shown to directly correlate with the degree of clinical severity of peri-implant disease (27). TNFα, among other direct and indirect effects, has a strong capacity to stimulate bone resorption by promoting cell differentiation from the monocyte-macrophage line to osteoclasts, inducing the maturation of osteoclasts and enhancing their resorptive activity (26,27).

Several studies (26) have shown a relationship between polymorphisms in the TNFα region (308-) and an increased susceptibility to various pathologies, including diabetes, coronary disease, retinopathy or polycystic ovarian syndrome. In relation to peri-implantitis, Rakic *et al.* (27) observed in a recent study of 369 Caucasian patients that the presence of the AG genotype in the polymorphic region of TNFα (308-) A/G was significantly higher in individuals with peri-implant pathology, with the positive patients displaying five times higher risk for peri-implantitis. In contrast, the GG genotype was much more prevalent in healthy individuals and statistically conferred a certain “protective effect”.

Jacobi-Gresser *et al.* (22), in a retrospective evaluation of 109 patients, studied the relationship between the risk of implant failure and the presence of polymorphisms in the IL-1 cluster and the TNF region: IL-1A (889-) C/T, IL-1B (+3954) T/C, IL-1RN (+2018) T/C and TNFα (308-) A/G. After the genetic analysis, they detected the presence of the AG genotype in the polymorphic region of TNFα (308-) in 46.3% of the patients who had suffered at least one implant failure and in 30.9% of control patients (OR = 1.9). They confirmed that subjects presenting a single polymorphism, from those described above, displayed a significantly higher risk of implant failure (OR = 1.57). They also observed an additive effect among the four polymorphisms, with carriers of all four alleles exhibiting the highest risk (OR = 6.01).

However, Campos *et al.* (28), analyzed the polymorphic region of TNFα (308-) A/G in a sample of 66 patients (28 patients who had suffered one or more failures and 38 controls, all non-smokers) and did not find any statistically significant relationship. Gurol *et al.* (29), in a study of 95 patients (16 with early implant failure, 22 with chronic periodontitis, 23 with healthy implants and 34 healthy controls), also found no significant relationship between TNFα (308-) A/G and an augmented risk of early failure. They also obtained negative results when analyzing specific polymorphisms of IL-10: IL-10 (1082-) A/G, IL-10 (819-) C/T and IL-10 (-592A/C).

Recently Mo *et al.* (30) performed a meta-analysis to clarify the controversial association between the SNP described above for TNFα and peri-implant pathology. Although they were unable to demonstrate a significant relationship, they suggested that further studies should be performed in larger patient samples to achieve definitive conclusions.

-Other polymorphisms

In the previously cited study by Rakic *et al.* (27) related to the polymorphism in the promoter region of TNFα (308-), the authors also analyzed the role of polymorphisms in the polymorphic region of CD-14 (159-) from the gene encoding the membrane receptor CD-14 (Cluster of Diferentiation-14). CD-14 allows monocytes, macrophages and polymorphonuclear cells to recognize lipopolysaccharides from the wall of Gram-negative bacteria. The results of this study demonstrated that the presence of the CC genotype was associated with a five times greater risk of peri-implantitis, while the CT genotype displayed a certain “protective effect”. They also found that carriers of the CC genotype had significantly higher concentrations of Receptor Activator of Nuclear Factor Kappa-Β Ligand (RANKL) in the peri-implant crevicular fluid.

RANKL is a key molecule for bone metabolism and is produced mainly by osteoblasts and fibroblasts. Upon binding of RANKL to its transmembrane receptor (RANK) it induces the resorptive capacity of osteoclasts. These activated osteoclasts subsequently acidify their environment, destabilizing the inorganic bone component and releasing enzymes such as matrix metalloproteinases (MMPs) or Cathepsin K, which in turn destroy organic components (31).

The MMPs are a family of 24 enzymes of similar structure but different genetic origin. They are divided into five different groups and are responsible for the degradation of the organic components of extracellular matrix, both in healthy homeostasis (i.e., continuous remodeling of tissues) and in disease (i.e., inflammatory processes). MMPs display diverse activities and functions, and are known to participate in tumor growth, the phenomena of metastasis and even cellular apoptosis. In addition to osteoclasts, various other cell types also produce MMPs, including fibroblasts, epithelial cells of the gingival sulcus, endothelial cells, plasma cells, monocytes/macrophages and neutrophils. Interestingly, even some oral microorganisms are capable of producing MMPs (MMP-1) (32). The physiological activity of MMPs is regulated by an intricate balance between their production and that of their antagonists, the TIMPs (Tissue Inhibitors of MMPs). This equilibrium has been found to be altered during various disease states (32). 

Different authors (33,34) have observed abnormally high concentrations of MMPs, especially MMP-8, in the saliva and crevicular fluid of patients with peri-implant disease. Notably, a strong clinical correlation between the degree of peri-implant pathology and the concentration of MMP-8 was also confirmed by these studies.

Costa-Junior *et al.* (35) analyzed the polymorphic region of MMP-8 (799-) C/T in 100 healthy controls and 80 patients who had suffered early failure of at least one implant. They verified that the presence of the GG genotype was statistically much more frequent (*p*= 0.0009) in the patient group with early failure. Therefore, they concluded that carriers of this genotype would have a higher risk of early failure.

In a group of 74 Brazilian patients (34 patients with early failure and 40 controls), Santos *et al.* (36) analyzed the polymorphic regions of MMP-1 (1607-) and MMP-9 (1562-) located in the promoter regions of these respective genes. Regarding the first SNP, they observed a higher frequency of a 2G allele, with an insertion of a second Guanine (5’-GGA-3’), among patients who had suffered early failure (50%, *p* = 0.013). With respect to the second SNP in MMP-9, they did not observe statistically significant differences.

The above researchers (37,38), in an analysis of the same patients, could not establish statistically significant relationships between early failure and other polymorphisms in the polymorphic regions of genes encoding Transforming Growth Factor beta 1 (TGFβ-1), IL-2 or IL-6: TGFβ-1 (509-) C/T, TGFβ-1 (800-), IL-2 (330-) or IL-6 (174-).

Casado *et al.* (39) in a sample of 103 patients (also of Brazilian origin; 52 healthy and 51 with peri-implant/periodontal pathology), found a positive relationship between the presence of a GG genotype in the IL-6 (174-) G/C region and risk of peri-implantitis. Therefore, it seems that the presence of the GG genotype could confer a greater risk of peri-implantitis (OR: 1.43¬ - 2.03).

In contrast, Melo *et al.* (40), studied the same polymorphic region of IL-6 in 47 Brazilian patients (31 healthy subjects and 16 patients with peri-implantitis) without finding any statistically significant relationship.

In addition to the above, other polymorphisms have also been analyzed and are summarized in  Table 1,  Table 1 continue.

**Table 1:**
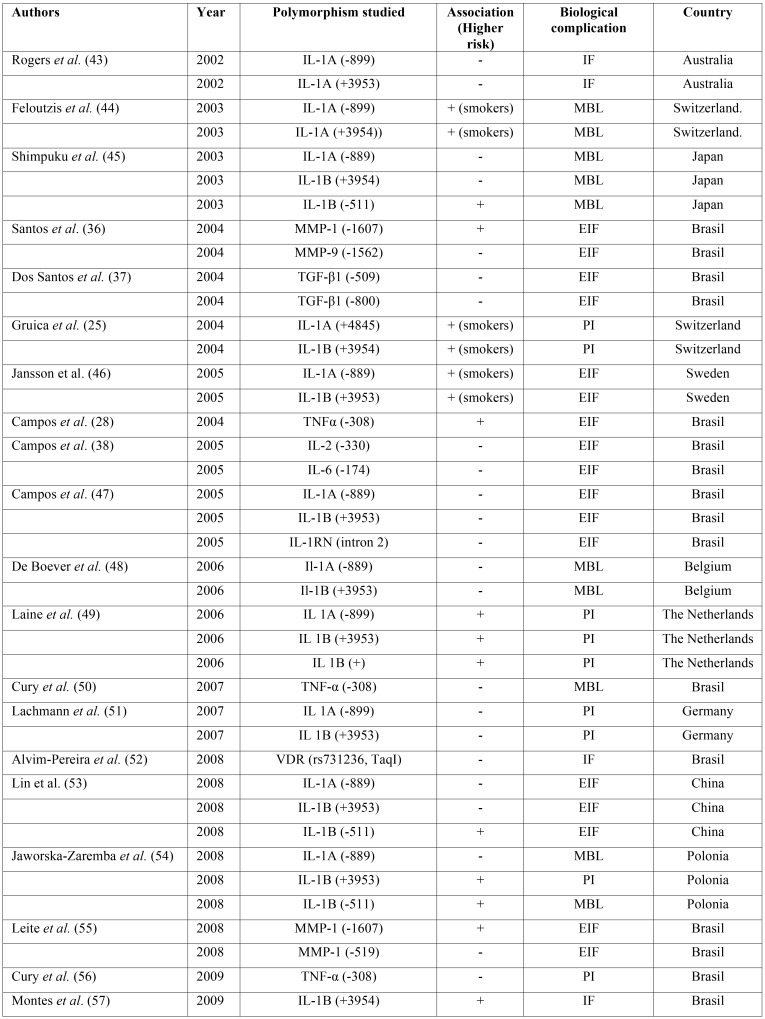
Summary of polymorphisms studied in relation to dental implants biological complications. IF: Implant loss, EIF: Early Implant Failure, PI: Peri-implantitis, MBL: Marginal Bone Loss.

**Table 1 continue:**
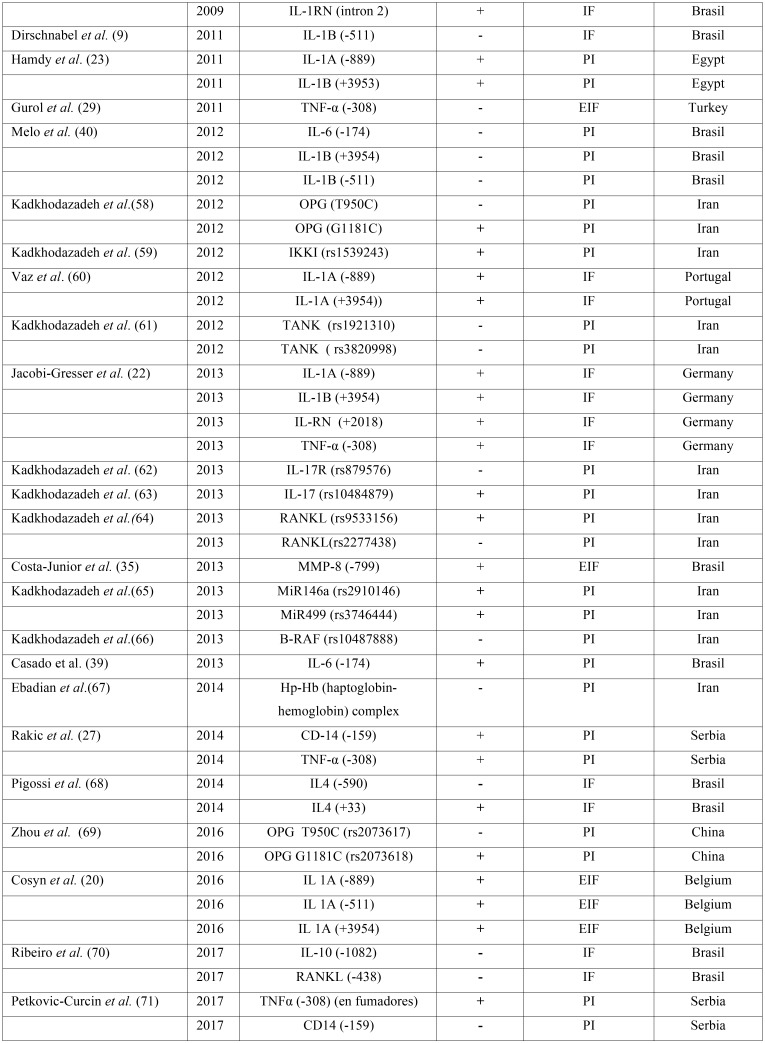
Summary of polymorphisms studied in relation to dental implants biological complications. IF: Implant loss, EIF: Early Implant Failure, PI: Peri-implantitis, MBL: Marginal Bone Loss.

## Discussion

In recent years, interest in the study of biomarkers in implantology has increased considerably, initially aimed at improving the predictability in the osseointegration phase of implants and more recently driven by a desire to reverse the increasing rates of peri-implantitis observed within the last decade (3,4). In view of the results that we have observed, we can confirm that this area of research currently remains in the developmental stages, with biomarkers mainly used experimentally. Although there is still no “commercial procedure” that allows routine clinical use, the promising initial results that have been published allow us to predict that in the future these biomarkers will be part of daily practice.

To date, the most developed biomarkers in implantology are the molecular and genetic biomarkers. The former is of great interest for the early diagnosis of peri-implantitis, not only to measure intensity and estimate activity, but also for the monitoring of both healthy and treated patients. In contrast, genetic biomarkers are developed for another purpose: to estimate the relative risk of an individual to suffer from peri-implantitis owing to their own innate characteristics. Irrespective of the presence of certain known risk factors associated with peri-implantitis, studies have tried to analyze statistically whether carrying certain “genetic peculiarities”, such as polymorphisms, could confer a higher relative risk of experiencing peri-implantitis (6-8).

Functionally relevant polymorphisms in genes that control expression of molecules regulating bone physiology and inflammatory response, for the most part, condition the overexpression of these genes. For this reason, they are also responsible for a dysregulated physiological response, especially under the action of known etiological factors for peri-implantitis. In line with this, these polymorphisms could then enhance the action of known risk factors, such as tobacco consumption, poor hygiene, the use of an inadequate prosthesis, etc. In example, polymorphisms might interact with the actions of known risk factors from a pathogenic point of view by contributing to breakage of the balance between the oral biofilm and the immune system, to disruption of the equilibrium between microfractures caused by occlusal forces transmitted to maxillary bones and the capacity for bone regeneration, or to increased corrosion of the implant surface layer. All of these phenomena are not fully understood and are surrounded by certain gaps in knowledge that require future clarification (6-8). 

Knowing in advance the relative “innate” risk of a patient to experience peri-implantitis, based on the detection of specific polymorphisms, would allow improvement or modification in the criteria for therapeutic indication. This information could also be used to “customize” and optimize monitoring and control protocols, or even make the treatment and therapeutic protocol selection more objective.

For most of the relevant articles available in the scientific literature a single polymorphism was analyzed. These studies attempted to standardize the samples and reduce possible biases, while examining in a statistically significant way, whether the presence of a specific genotype was more prevalent among individuals with biological complications compared to healthy patients. We found that the results are often contradictory between the different study groups. The specific methodologies utilized, and especially the criteria for patient selection, are critical points that might have resulted in such discrepancies. Several authors (10,21,24) have performed meta-analyses to try to shed light on the observed differences. These investigations identified that the number of studies available with a high level of scientific evidence is very small. In fact, many are preliminary studies with a low level of evidence or displaying methodological shortcomings, especially with regard to the uniformity of the study groups. Altogether, these factors make it difficult to come to definitive conclusions.

Many of the studies performed to analyze the relationship between biological complications in implantology and genetic polymorphisms are based on other similar investigations in relation to periodontitis (41). Undoubtedly, there are many similarities between natural teeth and implants, but there are also many aspects that are critically different, and of course these distinctions could specifically impact on this field. In this regard, a detailed review of the literature will allow us to verify how many of the relationships observed in periodontal pathology, either have not been found in studies on implantology or were not yet tested in relation to implants.

Another critical point that should be remembered when comparing studies and analyzing the importance of a certain genetic polymorphism, is that the ethnic origin of the study population must always be taken into account, since the prevalence of such polymorphisms in the general population can vary substantially. For example, some of the polymorphisms cited in relation to the IL-1 cluster are present in greater than one third of the population in Caucasians (41,42), but do not exceed 3% among Asian patients (42).

For advancing knowledge in this area of implantology, there is a fundamental need for new research. Among others, the main criticisms that we can draw from the meta-analyses and reviews existing in the literature in relation to some of the preliminary studies include: the shortage of prospective works, the reduced number of patients, the short or unspecified period of follow-up of patients or the lack of control with regard to systemic pathologies of interest (10,21,24). These points should be taken into account when designing future studies. Moreover, key points to consider in order to avoid biases should be the control of the ethnic origin of the patients as well as other risk factors experienced by the study group (not only tobacco consumption). Above all, although it requires an enormous effort, the sample homogenization should be tightly controlled (i.e., by type of treatment, types of implants, auxiliary surgical techniques used, location of implants, etc.).

In addition, given the high prevalence in the population of some of the polymorphisms cited throughout this work, it would be of great interest to study various polymorphisms in the same individual (considering a very large sample size) and not only analyze single polymorphisms. Although it is true that this would represent an enormous task, it could allow analysis of multivariable statistics and identify if there are summative or even synergistic effects between different types of polymorphisms.

Likewise, new studies are needed to measure the synergistic effect already observed by some authors (21,25) between polymorphisms and other risk factors, especially the consumption of tobacco.

An excellent option to discover new polymorphisms associated with peri-implant pathology, would be the realization of a Genome-Wide Association Study (GWAS) using large samples of multiethnic origin and “extreme genotypes”. Within these extreme genotypes it would be very interesting to include, among others, patients who have suffered what some authors have termed a “cluster failure”.

## Conclusions

There are few studies that have attempted to relate the presence of certain polymorphisms with a higher risk of early implant failure and/or peri-implantitis. The most studied polymorphisms are the SNPs. Among those that have shown a statistically significant relationship, there are some SNPs identified within the coding regions of genes, including mediators of inflammatory responses as well as molecules participating in physiological bone remodeling processes.

Based on available research and the current level of understanding regarding the relationship between polymorphisms for early failure or peri-implantitis, a reliable and/or reproducible genetic biomarker of risk has not yet been identified that could be used in the general population or commercialized. However, many authors agree that this is a field that warrants deeper investigation. Given the positive results of some preliminary investigations, new studies are needed that employ larger sample sizes and stringent methodologies in order to minimize the lack of uniformity and bias.
